# MRP4 over-expression has a role on both reducing nitric oxide-dependent antiplatelet effect and enhancing ADP induced platelet activation

**DOI:** 10.1007/s11239-020-02214-4

**Published:** 2020-08-14

**Authors:** Maria Luisa Guarino, Isabella Massimi, Laura Alemanno, Laura Conti, Dominick J. Angiolillo, Fabio M. Pulcinelli

**Affiliations:** 1 Department of Experimental Medicine, “Sapienza” - University of Rome, Viale Regina Elena 324, 00161 Rome, Italy; 2 Department of Research, Advanced Diagnostics and Technological Innovation IRCCS Regina Elena National Cancer Institute, Rome, Italy; 3 Division of Cardiology, University of Florida College of Medicine-Jacksonville, Jacksonville, FL USA

**Keywords:** Nitric oxide, Multidrug resistance protein 4, Aspirin, Platelet aggregation, Cilostazol

## Abstract

The impact of inhibition of multidrug resistance protein 4 (MRP4) on nitric oxide (NO) resistance and on ADP-induced platelet aggregation is unknown. The aim of this investigation was to verify whether platelet NO resistance correlates with MRP4 expression and evaluate whether this can be reduced by in vitro MRP4 inhibition mediated by cilostazol. Moreover, we assessed if inhibition of MRP4-mediated transport reduces ADP-induced platelet reactivity. The inhibitory effect of sodium nitroprusside (SNP), a NO-donor that enhances cyclic guanosine monophosphate (cGMP) cytosolic concentration, was assessed in platelets obtained from aspirin treated patients and in a control population. The inhibitory effect of SNP was evaluated by ADP-induced aggregation in SNP-treated platelets. The impact of MRP4 on ADP-induced platelet aggregation was performed in high on aspirin residual platelet reactivity (HARPR) patients and compared to healthy volunteers (HV), and a control cohort (CTR). In aspirin-treated patients with high levels of MRP4, reduced SNP inhibition was found compared to those with low levels of MRP4. MRP4 inhibition by cilostazol significantly reduced ADP-induced platelet aggregation in HARPR population, and to a lesser extent in HV and CTR populations. In conclusion, cilostazol can mitigate the hyper-reactive platelet phenotype of HARPR patients by reducing residual ADP-induced platelet aggregation and increasing NO-dependent endothelial antiplatelet effects.

**Electronic supplementary material:**

The online version of this article (10.1007/s11239-020-02214-4) contains supplementary material, which is available to authorized users.

## Highlights

- Platelets may have impaired physiological response to NO.
- MRP4 over-expression is associated with high on aspirin residual platelet reactivity.
- An association between platelet MRP4 overexpression and platelet NO resistance was found in subjects on chronic aspirin treatment.
- MRP4 inhibition induced by cilostazol or Ceefourin can mitigate the hyper-reactive platelet phenotype of HARPR patients by reducing residual ADP-induced platelet aggregation and increasing NO-dependent endothelial antiplatelet effects.

## Introduction

Platelets play a key role in haemostatic processes [1]. To limit excessive platelet activation and thrombus formation, platelets are regulated by endothelial cell-derived inhibitory signals such as nitric oxide (NO). NO inhibits platelet aggregation by increasing cyclic guanosine monophosphate (cGMP) levels [2] leading to protein kinases G (PKG) activation that phosphorylates a variety of substrates, including vasodilator-stimulated phosphoprotein (VASP) [3]. Although the “tissue NO resistance” phenomenon, defined as an impaired physiological response to endogenous and exogenous NO, has been shown, its mechanism(s) remain unclear [4]. Sassi et al. demonstrated secretion of cyclic nucleotides from the cytoplasm through the multidrug-resistance protein MRP4/ABCC4 [5]. In human platelets, MRP4 is mainly located on the membrane of dense granules and to a lesser extent on the plasma membrane [6] where it acts as a negative regulator of cyclic nucleotides hens limiting their inhibitory effects [6, 7]. The role of MRP4 in modulating platelet function by regulating intracellular concentration of cyclic nucleotides was demonstrated in a MRP4 knock-out mouse model showing prolonged bleeding times, defective platelet aggregation and in vivo thrombus formation [8, 9]. Most recently, impaired platelet aggregation in response to ADP was observed in ABCC4 null individuals [10].

**Fig. 1:**
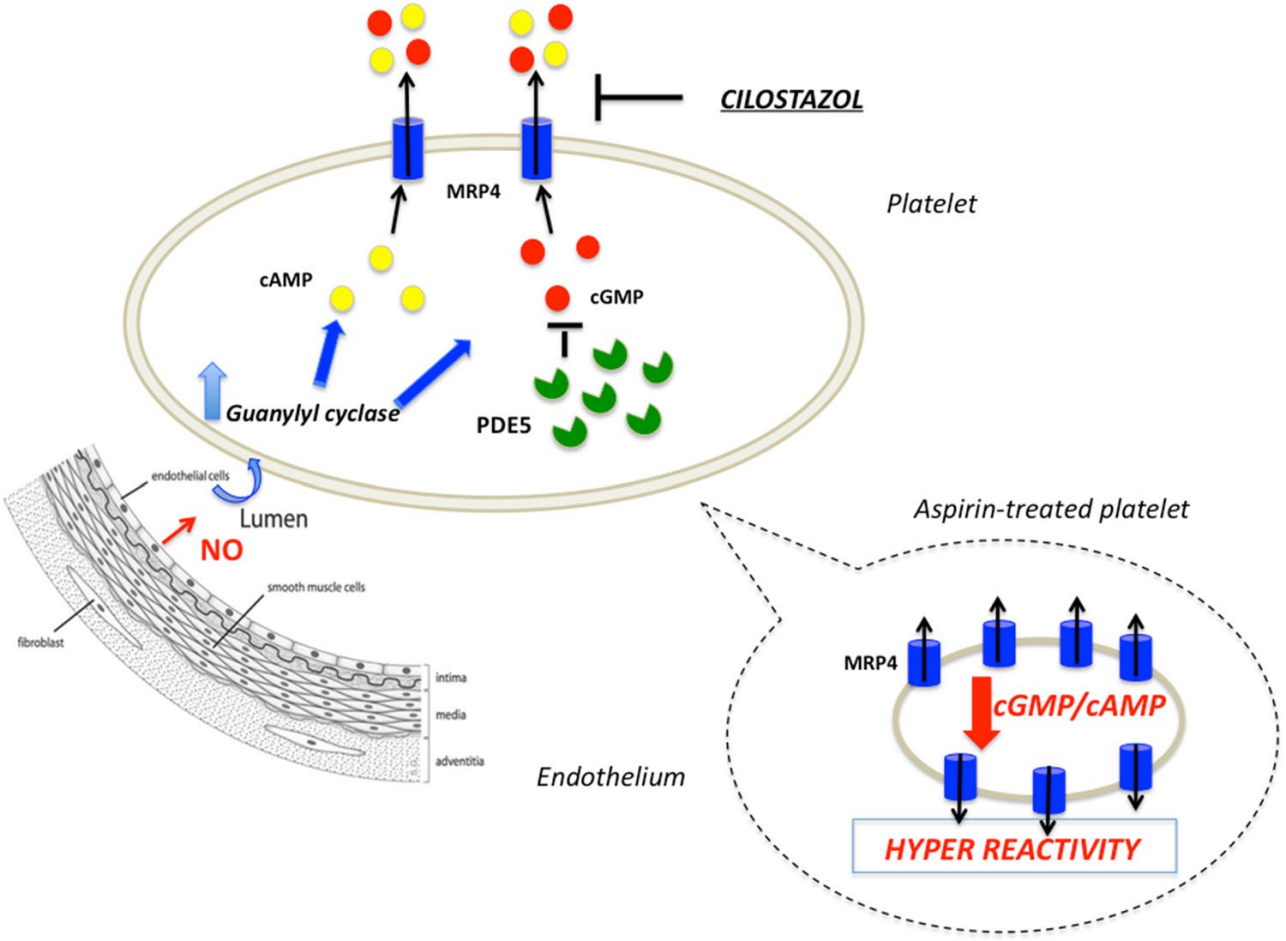
Interaction between platelets and endothelium and the role supposed by MRP4 over-expression in aspirin-treated platelets

Platelets with MRP4 overexpression, such as those obtained from subjects with human immunodeficiency virus (HIV), have reduced cytosolic cAMP levels [11]. Moreover, increased platelet levels of ABCC4-mRNA identifies a hyperreactive platelet phenotype, which has been suggested to contribute to HIV-mediated cardiovascular disease [11]. Our group demonstrated that MRP4 can be up-regulated in human platelets by aspirin [12–14] and that platelets with MRP4 overexpression are more reactive to agonists [15]. Moreover, in patients on chronic aspirin treatment, we found a correlation between platelet MRP4 levels and high on-aspirin residual platelet reactivity (HARPR) [16]. Recently we reported that inhibiting MRP4 using cilostazol decreases platelet function and HARPR without enhancing cytosolic cAMP concentration [16]. Figure 1 reported the interaction between platelet and endothelium and the role of MRP4 in reducing cyclic nucleotides action.

The impact of cilostazol-induced MRP4 inhibition on NO resistance is unknown. The aim of this investigation was to verify whether platelet NO resistance correlates with MRP4 expression and evaluate whether this can be reduced by in vitro MRP4 inhibition mediated by cilostazol. Moreover, we assessed if inhibition of MRP4-mediated transport reduces ADP-induced platelet reactivity.

## Materials and methods

The experimental design of our investigation was carried out in several phases:

- We studied the effects of MRP4 on NO resistance and evaluated whether this is affected by MRP4 inhibition induced by cilostazol;
- We determined the time at which cilostazol is only a MRP4 inhibitor in NO treated platelets without enhancing the cyclic nucleotide inhibitory effect.
- The impact of MRP4 on ADP-induced platelet aggregation was studied by adding agonists following cilostazol and Ceefourin treatment.

These experiments were conducted in platelets obtained from three separate cohorts of subjects: (a) healthy volunteers (HV), (b) patients on chronic aspirin treatment (ASA) and (c) a control cohort (CTR). The ASA and CTR populations were similar for risk factors and medications, except for aspirin assumption.

### Study population

- HV population: included non-medicated volunteers, without a history of coronary heart disease or risk factors for atherothrombosis.
- ASA population: included patients with a history of coronary heart disease or with one or more risk factors for atherothrombosis on aspirin treatment (100 mg daily) for at least 2 months (ASA) as part of their standard of care. Compliance was assessed by interview and confirmed by arachidonic acid-induced platelet aggregation (0.75 mM) < 10%.
- CTR population: included patients with risk factors and medications similar to the ASA population, except for aspirin assumption.

Platelets from the HV (n = 53) and ASA (n = 62) populations were used to study NO resistance. Platelets from the HV (n = 20), CTR (n = 18) and HARPR (n = 38) population, defined as patients under chronic aspirin treatment with more than 40% of aggregation to collagen 4 μg/ml [16] were used to study MRP4 and ADP-induced platelet aggregation.

This study was carried out according to the Declaration of Helsinki. All study participants gave written informed consent.

### Blood sampling and laboratory assessments

Blood was collected in sodium citrate 3.8% and platelet-rich plasma (PRP) was obtained by centrifugation at 200×*g* for 15 min. Plasma-free platelets (PFPs) were obtained by centrifugation of PRP in presence of 10% of ACD (2.5% sodium citrate, 1.5% citric acid, 2% glucose) at 10,000×*g* for 2 min and resuspended in Tyrode’s buffer at pH 7.35 (NaCl 136 mM, KCl 2.7 mM, HEPES 10 mM and 0.1% glucose) [17]. For RNA extraction, platelet pellet was re-suspended with Tyrode’s buffer in presence of EDTA 5 mM and filtered through a 5 µm syringe-adaptable filter to remove white and red blood cells contaminants.

### Platelet aggregation

Platelet aggregation (PA) was performed as previously described [18]. PA was evaluated both in PRP and PFP using AggrRam aggregometer (Helena Laboratries, Beaumont, Texas, USA). To study NO resistance in platelets, we evaluated ADP (10 µM, Helena Bioscience, Tyne and Wear, UK) induced aggregation in platelets pre-treated with sodium nitroprusside (SNP), a NO donor, in the ASA population. Aspirin (10 µM, 30 min) treated platelets were incubated with SNP (50 µM, Sigma-Aldrich Corporation, Saint Louis, Missouri, United States) for 1 min before agonist addition. Arachidonic acid 0.75 mM (Helena Bioscience) induced-PA was performed to assess aspirin efficacy. In some experiments we also evaluated PA in the presence of cilostazol (20 µM; Italfarmaco spa, Italy) and Ceefourin-1 (50 µM; Abcam, Cambridge, UK) in which the agonist was added 10 s after drug treatment. Results are reported as the percentage of platelet aggregation (%PA) observed after 4 min of agonist stimulation. To study the role of MRP4 on ADP-induced PA we performed aggregation studies in aspirinated PFPs either in absence or in presence of the MRP4 inhibitors, cilostazol (5 µM for 10 s) and Ceefourin 1 (20 µM for 10 s) using 2 µM and 5 µM of ADP.

### MRP4-mRNA determination

Total platelet RNA was extracted using TRIzol reagent (Invitrogen, San Diego, CA).

For mRNA detection 1 μg of total RNA was transcribed using the GeneAmp Gold RNA PCR Reagent Kit pAW109 (Applied Biosystems, Warrington, UK) according to the manufacturer's suggestion. Gene expression was performed using Q-RT-PCR with TaqMan Master Mix and TaqMan Assay Reagent (Life Technologies) [19]. Changes in MRP4, CXCL8 and ACTIN-mRNA amounts were quantified by ΔΔCt method for relative quantization of gene expression using SDS software version 2.3 (Applied Biosystems). All samples were free of mature transcript for CXCL8 (Interleukin-8), a leukocyte-specific gene product, to support that absence of leukocyte contamination [12].

### VASP phosphorylation studies

VASP is a substrate of PKG that is activated by cGMP. To evaluate the effects of cilostazol on increasing cGMP cytosolic concentration we studied VASP phosphorylation in SNP-treated platelets. The effect of cilostazol on Ser-239 VASP phosphorylation was assessed at different point time (T-10″, T-60″, T-300″) after 0SNP treatment in aspirinated PFPs (3 × 10^5^ cells/ µl) according to [16]. Platelet lysates were separated by SDS polyacrylamide electrophoresis (Bio Rad, California, United States) and transferred to polyvinylidene fluoride membrane (Ge Healthcare, Milano, Italy). Phosphorylated VASP was detected using phosphor-VASP (Ser-239) mouse antibody (Alexis Biochemicals, Lausen, Switzerland). Primary antibody was detected using a peroxidase-coniugated anti-mouse antibody and ECL (Perkin Elmer). Equal loading amount of proteins is represented by total VASP expression (Alexis Biochemicals).

### Statistical analysis

Categorical variables are expressed as frequencies and percentages. Continuous variables following a normal distribution are expressed as mean ± standard deviation. Comparison between normally distributed continuous variables were performed using Student’s t-test for unpaired and paired data. Variables not following a normal distribution are illustrated as a BOX plot graph, which shows the distribution around the median of the populations. Comparisons for data not normally distributed were evaluated by Wilcoxon test. Results were considered statistically significant if a p-value of less than 0.05 was reached. Statistical analysis was performed using a KaleidaGraph software 3.6 (Synergy Software, Reading, Pennsylvania, USA).

## Results

### Correlation between NO resistance and MRP4 expression

**Fig. 2:**
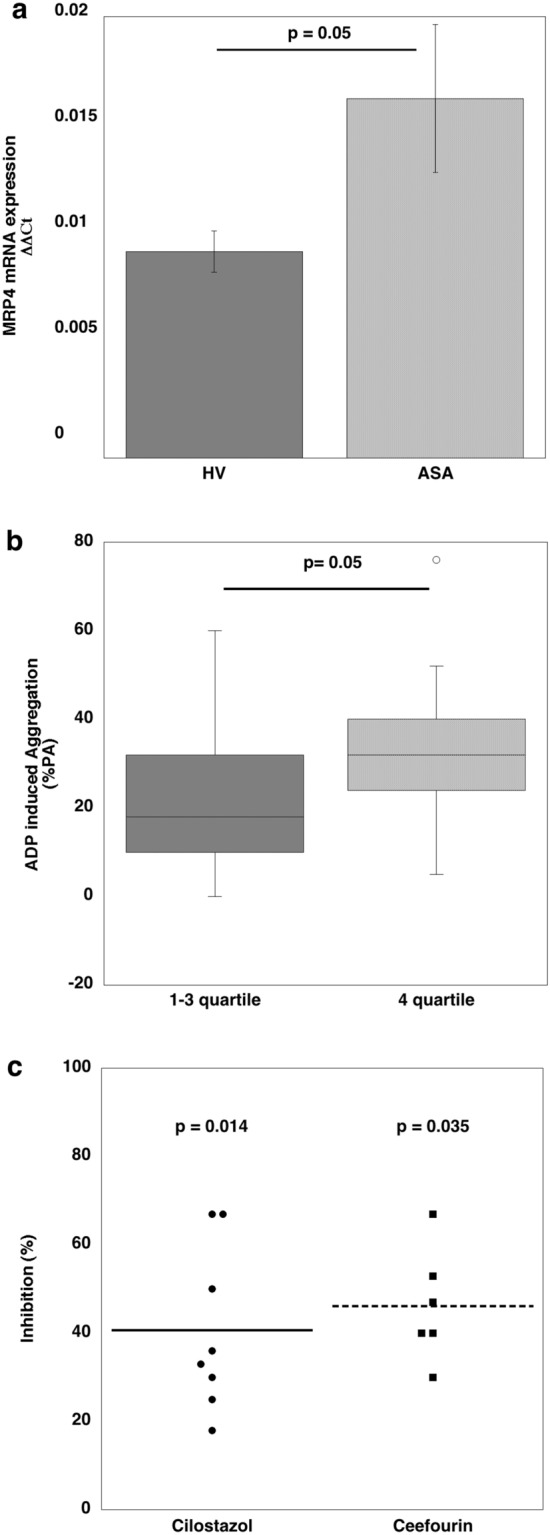
**a** Histogram of Q-RT-PCR analysis of MRP4 mRNA expression in aspirin treated patients (ASA > 2 months N = 52) and healthy volunteers (N = 38). Data were normalized with actin expression and reported as mean ± SD of fold increase. Statistical data was evaluated by Student’s t-test for unpaired samples. **b** Box plot of platelet aggregation induced by ADP (10 μM) in SNP (50 μM) treated platelets obtained from aspirinated patients belonging to the 1st to 3rd quartiles (N = 39) versus patients belonging to the 4th quartile (N = 13) for MRP4 expression levels. Platelet aggregation is reported as percentage evaluated 4 min (PA%). Statistical data was evaluated by Wilcoxon test for unpaired samples. ADP aggregation in platelets untreated with SNP was 81 ± 6%. **c** Enhancement of SNP (50 μM) inhibitory effect of Cilostazol (20 μM) and Ceefourin **(**50 μM) on ADP (10 μM) induced platelet aggregation in chronic aspirin treated patients with ADP-induced aggregation > 20%. Cilostazol and Ceefourin were added to SNP treated platelets 10 s before agonist addition. The data are expressed as a percentage of inhibition in comparison with SNP treatment of each sample. Statistical difference was evaluated by Wilcoxon-test for paired samples. ADP induced aggregation in SNP treated platelets was 22 ± 16%

The ASA population was characterized by over expression of MRP4-mRNA compared to HV (0.016 ± 0.003 vs 0.009 ± 0.0009; p = 0.05) (Fig. 2a). We subsequently evaluated whether in ASA patients SNP reduces ADP-induced PA and we obtained that this was reduced by 75% (81 ± 6% vs 22 ± 16%). In order to evaluate the correlation between MRP4 levels and SNP depended platelet inhibition, we divided the ASA population into quartiles for MRP4 expression. The first group consisted of patients, belonging to the 1st-3rd quartiles (MRP4-mRNA values from 0.005 to 0.014 ΔΔCt); the second group, consisting of patients belonging to the 4th quartile (MRP4-mRNA values from 0.015 to 0.031 ΔΔCt). Patients with high levels of MRP4 (ΔΔCt > 0.015) had increased PA and reduced SNP inhibition compared to those with low levels of MRP4 (ΔΔCt < 0.015) (32 ± 18% vs 22 ± 15%; p = 0.05) (Fig. 2b). In order to confirm a role of MRP4 in reducing SNP effects, platelets obtained from 8 patients with ADP-induced PA > 20% after SNP treatment were incubated with two MRP4 inhibitors cilostazol (20 μM) and Ceefourin 1 (20 μM) for 10 s before addition of the agonist. Such treatment enhanced NO inhibition by approximately 60% (Fig. 2c).

Representative tracer are reported in Supplementary Material (Fig. S1).

### Effect of cilostazol on VASP phosphorylation

To discriminate if the effects of cilostazol were due to the Phosphodiesterase (PDE) inhibition or to MRP4 inhibition, we studied a time depended cilostazol effect on VASP phosphorylation (Serine-239), in SNP-treated platelets. After 10 s, cilostazol had no effect on the enhancement of VASP phosphorylation. However, we observed an increase in VASP phosphorylation at T-60″ and after 5 min (T-300″), suggesting that at this incubation time there is an increase of cGMP cytosolic concentration. This result shows that after 10 s of treatment, the inhibitory effect of cilostazol is not due to the enhancement of cGMP cytosolic concentration (Fig. 2 Supplementary).

### MRP4 and ADP-induced platelet activation

To study the effects of ADP, without the interference of thromboxane-A2 production, platelets obtained from HV, CTR and HARPR subjects were treated in vitro with aspirin 10 µM. PA was induced using ADP. At low ADP concentration (2 μM), aspirin treatment in the HARPR population slightly increased PA (59 ± 22% vs 43 ± 28%; p = 0.006), while aspirin treatment reduced ADP-induced PA in the CTR (41 ± 18% vs 75 ± 28%; p = 0.002) and HV (24 ± 10% vs 57 ± 40% p = 0.021) populations.

**Fig. 3:**
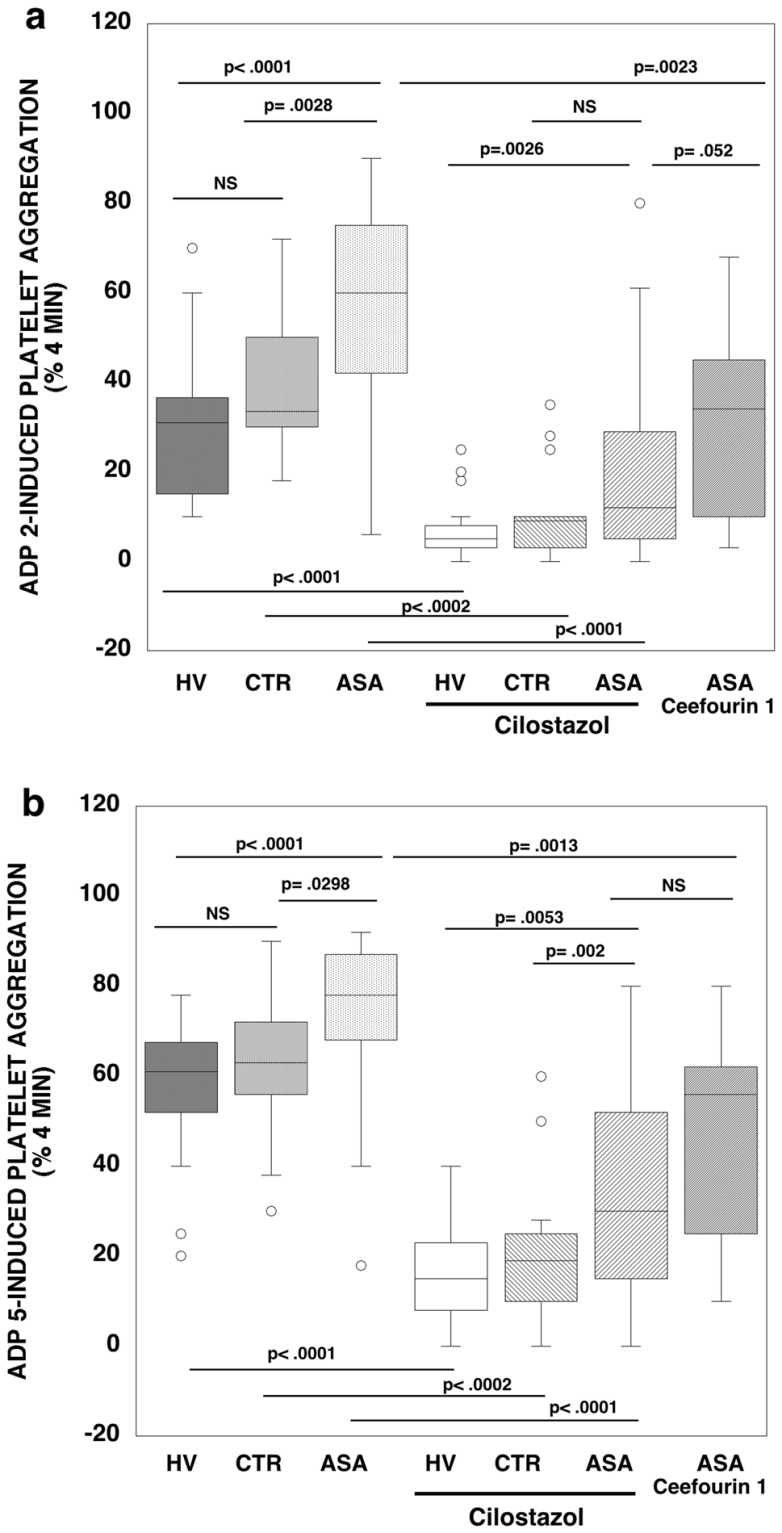
Box plot of ADP 2 μM (**a**) and 5 μM (**b**)-induced platelet aggregation in healthy volunteers (HV; N = 20) control popuation (CTR; N = 18) and HARPR patients (ASA; N = 38) in-vitro treated with aspirin 10 μM, with and without Cilostazol (5 μM) or Ceefourin (20 μM; N = 10). Aggregation of platelet free plasma is reported as percentage measured 4 min (%PA). Data are expressed as the mean ± SD. Statistical data was evaluated by Wilcoxon test for unpaired samples for the differences between the population and for paired samples between untreated and cilostazol or Ceefourin-treated population

The MRP4 inhibitor, cilostazol, significantly reduced ADP (2 μM)-induced PA in the ASA (19 ± 18% vs 58 ± 21% p < 0.0001), HV (7 ± 6% vs 23 ± 10%, p < 0.0001) and CTR (11 ± 10% vs 40 ± 17%, p < 0.0002) populations (Fig. 3a). Using a higher ADP concentration (5 μM), the difference of PA, after aspirin treatment, is less evident between the HV, CTR and ASA populations and cilostazol treatment still significantly reduces platelet aggregation in the ASA (34 ± 22% vs 73 ± 16%, p < 0.0001), HV (16 ± 10% vs 58 ± 15% p < 0.0001) and CTR (20 ± 15% vs 64 ± 16% vs p < 0.0002) populations (Fig. 3b). Ceefourin-1, a selective inhibitor of MRP4, reduces ADP-induced PA in aspirin treated PFPs at both 2 μM and 5 μM (32 ± 20% vs 58 ± 21% p = 0.0023 and 48 ± 23% vs 73 ± 16% p = 0.0013 respectively).

## Discussion

NO plays an essential role in the process of haemostasis by enhancing cytosolic concentration of cGMP and hence inhibiting platelet activation and thrombus formation [20]. The phenomenon of “tissue NO resistance” is defined as an impaired physiological response to NO [4]. NO resistance may therefore be associated with an increased risk of thrombotic events [4]. We previously demonstrated that MRP4 is a negative endogenous regulator of cyclic nucleotide intracellular levels in human platelets and therefore leads to reduction of their inhibitory effects [7]. In this investigation we demonstrated that in platelets obtained in aspirin treatment patients with MRP4 overexpression such defense mechanism is decreased. Moreover, by reducing MRP4 transport using an inhibitor (i.e., cilostazol) such protective mechanism is restored. We report an association between platelet MRP4 overexpression and platelet NO resistance found in subjects on chronic aspirin treatment. In particular, aspirin treated patients with high levels of MRP4 (ΔΔCt > 0.015) are less sensitive to SNP (a NO donor) inhibition compared to patients with lower levels of platelet MRP4.

The inhibition of MRP4-mediated transport enhances SNP-induced platelet inhibition even in a cGMP independent manner. In fact, cilostazol treatment is enough for reduces ADP-induced aggregation in SNP-treated platelets even at 10 s before the addition of the agonist. At that time of incubation, cilostazol is not able to enhance VASP phosphorylation induced by SNP. This data suggests that reduced aggragation is depended only on MRP4 inhibition. MRP4 role in platelet function has been demonstrated in MRP4 knockout mice and in null PEL patients in which the lack of the transporter in platelets determines reduced platelet functionality [9] Azouzi (2020).

The capability of cilostazol to enhance SNP inhibitor effect can be ascribed to MRP4 inhibition as Ceefourin, a selective MRP4 inhibitor, showed similar enhancement of the SNP inhibitory effect. We can assume that a platelet phenotype with MRP4 overexpression in patients taking aspirin shows reduced endothelial inhibitory effects [13]. These observations can potentially lead to an increased risk of thrombotic complications.

In our investigation we also showed that platelets treated in vitro with aspirin had increased platelet activity using ADP at a low concentration (2 μM) in an HARPR population compared to HV and CTR populations. Cilostazol treatment reduces ADP-induced PA in all populations. By increasing ADP concentration (5 μM), the difference in PA is reduced in the 3 populations (HV, CTR, and ASA) after in vitro aspirin treatment. However, cilostazol is still able to reduce such aggregation. This means that platelets obtained from HARPR patients are more sensitive to ADP. Cilostazol’s efficacy on reducing ADP-induced PA is only MRP4 dependent and not secondary to residual TxA2 production, as we found that with in vitro aspirin treatment (10 μM) COX-1 is almost completely inhibited leading to lack of TxB2 production [13]. Similar inhibition was found using Ceefourin, a selective MRP4 inhibitor. These results are in accordance to Decouture et al. who demonstrated that at low ADP concentrations (2 μM) in an MRP4 knock-out mouse, PA was significantly lower than those found in wild-type mice, whereas, there were no differences at a higher ADP concentration (10 μM) [8] and to those from Azouzi et al. showing impaired PA at low ADP concentration in ABCC4 null individuals [10].

Our group recently demonstrated a correlation between MRP4 overexpression and P2Y1 overexpression in platelets [21]. Our findings suggest that both the enhancement of P2Y1 receptors and MRP4 overexpression in platelets are involved in HARPR. In fact, both cilostazol and Ceefourin, after 10 s of incubation reduces ADP-induced PA. The efficacy of cilostazol to enhance antiplatelet effects was also demonstrated in vivo among patients undergoing percutaneous coronary intervention treated with standard dual antiplatelet therapy with aspirin and clopidogrel showing a reduction in platelet reactivity as well as a reduction in atherothrombotic events with the adjunctive use of cilostazol [22–24]. Recently, a potential role for adjunctive cilostazol therapy was suggested for patients at high-risk non-cardioembolic ischaemic stroke [25, 26]. Of note, in these studies such enhanced efficacy associated with adjunctive cilostazol therapy occurred without any increase in bleeding complications. Overall, our results indicate that cilostazol, in addition to reducing collagen, TRAP [16] and ADP platelet reactivity shown in this paper, also enhances the endothelial derived inhibitory effect on circulating platelets. Therefore, the effect of cilostazol on MRP4 function may be another mechanism that can explain the benefits of adjunctive treatment with cilostazol on reducing atherothrombotic events.

In conclusion, subjects with platelet MRP4 overexpression are less sensitive to NO protection. Cilostazol can mitigate the hyper-reactive platelet phenotype of these subjects by reducing residual ADP-induced PA and increasing NO-dependent endothelial antiplatelet effects. This treatment may represent a novel antithrombotic strategy to reduce cardiovascular events, particularly among patients less sensitive to aspirin due to MRP4 platelet overexpression.

## Electronic supplementary material

Below is the link to the electronic supplementary material.

Supplementary file1 (PDF 900 kb)
