# Supplementary material for: MRP4 over-expression has a role on both reducing nitric oxide-dependent antiplatelet effect and enhancing ADP induced platelet activation
Source: J Thromb Thrombolysis. 2020 Aug 14;51(3):625–32. doi: 10.1007/s11239-020-02214-4 (PMC8049923; doi:10.1007/s11239-020-02214-4)
Supplement: Supplementary file 1 — Supplementary file1 (PDF 900 kb) [file 11239_2020_2214_MOESM1_ESM.pdf]

Supplementary Materials

Figure S1

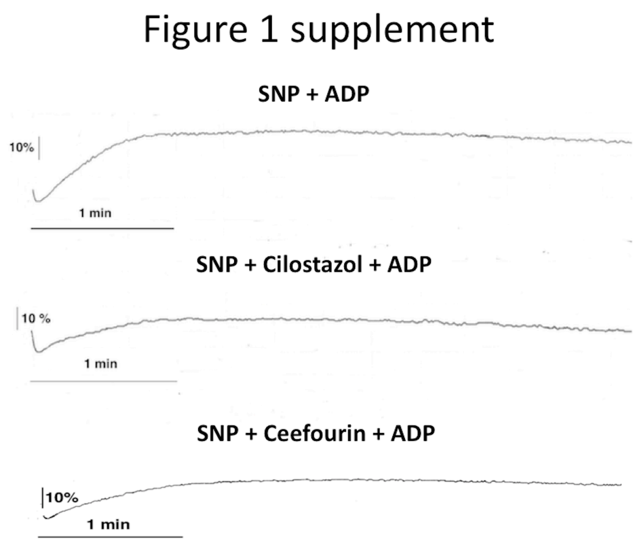

Representative tracers of the effect of Cilostazol (20 μM) and Ceefourin (50 μM) on ADP (10 μM) induced platelet aggregation in chronic aspirin treated patients with ADP-induced aggregation >20%. Cilostazol and Ceefourin were added to SNP treated platelets 10 sec before agonist addition.

Figure S2

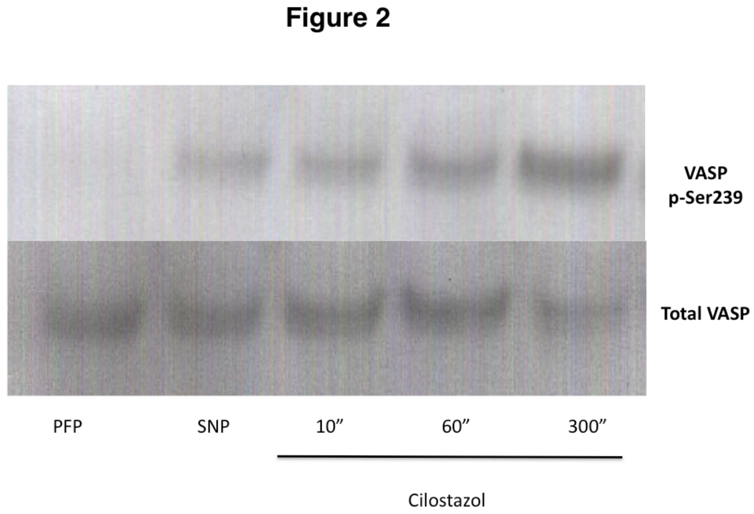

Western blot of 2 performed p-VASP(p-239) in platelets free plasma (PFP) and in SNP-treated platelets untreated and treated with Cilostazol (20 μM) for 10-30-300 sec, to evaluate the expression of c-AMP activity. Equal loading amount of proteins is represented by total VASP expression.
